# Twelve-Week Aerobic Training Decreases Chemerin Level and Improves Cardiometabolic Risk Factors in Overweight and Obese Men

**DOI:** 10.5812/asjsm.34860

**Published:** 2010-09

**Authors:** Abbas Saremi, Nader Shavandi, Mohammad Parastesh, Hassan Daneshmand

**Affiliations:** 1 Department of Sport Sciences, Arak University, Arak, IR Iran; 2 Department of Medical Sciences, University of Arak, Arak, IR Iran

**Keywords:** Exercise, Training, Obesity, Metabolic syndrome, Chemerin

## Abstract

**Purpose:**

The inflammatory state of adipose tissue in obese subjects may be the most important factor linking increased adipose tissue mass to insulin resistance. Chemerin is a newly discovered adipokine that plays an important role in macrophage infiltration into adipose tissue and may contribute to the development of inflammation and insulin resistance. We examined the effects of 12 weeks of aerobic training on serum chemerin levels in association with cardiovascular risk factors in overweight and obese males.

**Methods:**

Twenty-one overweight and obese subjects [44.3 (±4.1 yrs, body mass index (BMI) ≥25 kg/m^2^) were assigned to exercise training (obese EX, n= 11) and control (obese CON, n= 10) groups. The obese EX group participated in 12 weeks of progressive aerobic training 5 days a week. Serum chemerin, insulin resistance, lipid profiles, blood pressure, and body composition were all measured before and after the training.

**Results:**

After the aerobic training, waist circumference (*P*=0.009), fat percent (*P*=0.03), visceral fat (*P*=0.03), subcutaneous fat (*P*=0.01), fasting glucose (*P*=0.01), insulin resistance (*P*=0.03), triglyceride (*P*=0.05), total cholesterol (*P*=0.04), low-density lipoprotein cholesterol (*P*=0.05) and systolic blood pressure (*P*=0.04) of participates were significantly decreased. Concurrently, serum chemerin concentrations were significantly decreased after aerobic program (*P*=0.02).

**Conclusion:**

Aerobic training caused an improvement in cardiometabolic risk factors in obese subjects, and this improvement was accompanied by decreased chemerin levels.

## INTRODUCTION

Obesity, particularly visceral adiposity, is accompanied by chronic low-grade inflammation, indicated by increased serum levels of inflammatory markers such as C-reactive protein (CRP), tumor necrosis factor-α (TNF-α), and interleukin-6 (IL-6) of obese patients^[1, 2]^. Chronic inflammation is a well-known risk factor of insulin resistance and metabolic syndrome^[3]^. It is now generally considered that macrophages infiltrating into obese adipose tissue from circulation are a primary source of inflammation in obesity^[4]^ and could be involved in pathophysiology of insulin resistance ^[5]^. Cellular and molecular mechanisms responsible for this macrophage infiltration are not clear.

Recently, it has been demonstrated that chemerin may play an important role in macrophage infiltration into adipose tissue^[6–8]^. Chemerin is a novel adipokine that has been reported to serve as a chemoattractant for macrophages via direct interaction with its receptors (chemokine-like receptor-1)^[6–8]^. It has been reported that chemerin expression in adipose tissue is increased in obese mice^[9]^. In addition, circulating levels of chemerin are elevated in obese and diabetics subjects ^[10]^. Furthermore, in vitro studies revealed that treatment with chemerin induces insulin resistance in human skeletal muscle cells at the levels of IRS1 (Insulin receptor substrate 1), protein kinases B (PKB), and Glycogen synthase kinase 3 (GSK3) phosphorrylation and glucose uptake^[11]^. Several studies have investigated the association between metabolic syndrome indices and chemerin^[10–12]^. In these studies chemerin levels were positively correlated with body mass index, waist circumference, blood pressure, triglyceride (TG), low-density lipoprotein cholesterol (LDL-C) levels and insulin resistance, and were inversely correlated with adiponectin and high-density lipoprotein cholesterol (HDL-C) levels. Taken together, these findings suggest that chemerin play an important role in macrophage infiltration into adipose tissue and may contribute to development of inflammation and insulin resistance.

Physical inactivity is a well known risk factor of type2 diabetes mellitus development ^[13]^ and aerobic training has been shown to reduce adiposity and insulin resistance in obese adults^[14]^. While we know a lot about the mechanisms by which adiposity leads to insulin resistance^[15]^ and how exercise increases insulin sensitivity^[16, 17]^, it hasn't been yet reported about the exercise-induced changes in chemerin concentrations, which may provide a link between obesity and insulin resistance. Therefore, the aim of this study was to assess the effect of aerobic training on serum chemerin in relation to improvement of insulin sensitivity in overweight and obese men.

## METHODS AND SUBJECTS

**Fig. 1:**
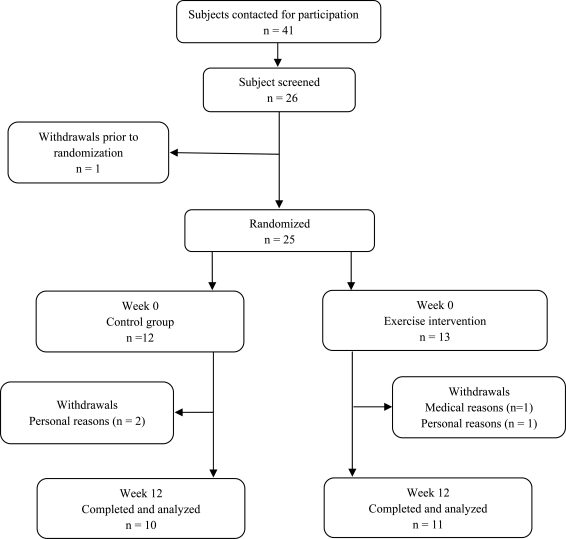
Flow chart of subject participation.

Twenty-one overweight and obese, middle-aged and sedentary men [age 44.3 (±4.1) yrs, body mass index (BMI)≥25 kg/m^2^] were recruited to the present study. All participants underwent an initial eligibility screening in exercise physiology laboratory of Arak University. Eligible participants were randomized to exercise training (obese EX, n=11) and control (obese CON, n=10) groups. Figure 1 presents the distribution of study participants. The obese EX group participated in 12-week aerobic training program, while the obese CON maintained their lifestyle as usual. All subjects had a stable weight for at least 3 months before inclusion. We excluded candidates who were smoker, had cardiovascular diseases or any other major illnesses, or were taking medications that could affect laboratory test results. The physical activity level of subjects was assessed during an interview. The ethics committee of university approved the experimental procedures and study protocols, which were fully explained to all subjects. A written consent form was signed by each subject after having read and understood details of the experiments.

**Dietary assessment:** The subjects were instructed to follow their usual eating habits during the course of the study. Dietary intakes were registered during the 12-wk intervention period using 3-d dietary records. The participants were provided with several practice sessions regarding a measuring cup, spoon and ruled paper until they learned how to estimate the sizes of food items consumed. Then food records were analyzed using the food processor II nutrition system-analysis software Version 3.1 (Tehran University of Medical Sciences, Iran) to determine total daily caloric and macronutrient intake.

**Aerobic training program:** Aerobic training supervised by an exercise physiologist was performed 50–60 min/d, 5d/wk, for 12 weeks. At each training session, those in the exercise group performed warm- up exercises lasting 10 min, followed by a 15–50 min walking-running, and 10 min of relaxation exercises at the end of the exercise period. Exercise intensity progressed from 15-20 min at 60–65% of HR_max_ during the first week to 25–30 min at 60–70% of HR_max_ by week 3, 35–40 min at 75–80% of HR_max_ by week 7 and 45–50 min at 80–85% of HR_max_ by week 12. Target heart rates were monitored using telemetry. Aerobic training included treadmill walking/running. Peak oxygen consumption (VO_2_ peak) was measured using a graded treadmill test and online computer-assisted open-circuit spirometry. Peak VO_2_ was attained when subjects achieved at least two of the following criteria: (a) a respiratory exchange ratio in excess of 1.0, (b) reaching ≥85% of age-predicted HR_max_, or (c) achieving a rating of perceived exertion (Borg Scale) ≥17.

**Anthropometric and body composition measurements:** Height (m) and weight (kg) were measured to calculate BMI as body weight (kg)/height^2^ (m). Waist circumference was measured at the narrowest point superior to the hip using a tape ruler. Whole body fat mass and percent body fat was assessed by bioelectric impedance analysis (Body composition analyzer, In Body 3.0, Korea). A single-slice computed tomography scan taken midway between L4 and L5 was performed using a Siemens Helicoidal Somatom Balance Scanner (Siemens, Erlangen, Germany) to measure visceral and subcutaneous adipose tissue areas as previously described^[18]^.

**Blood pressure and blood sample analysis:** Blood pressure was measured twice, after a 10-min rest with a random zero mercury sphygmomanometer, and was averaged. All subjects reported for blood sampling in the morning after an overnight fast. Post- training blood samples (10 ml) from subjects in the training group were obtained 48 h after their last exercise session. After collection, blood samples were centrifuged (40c, 1500×g, 15min) and plasma- and serum-containing tubes were stored at −70°c until analysis. Plasma total cholesterol, triglyceride, and HDL-C were measured by enzymatic colorimetric methods (Pars Azmun, Tehran, Iran). LDL-C was calculated using the formula of Friedewald. Plasma glucose was determined by the glucose oxidase method (Pars Azmun, Tehran, Iran). Plasma insulin was determined by chemiluminescent immunometric assay (Monobind Inc, USA). The homeostasis model assessment (HOMA), insulin resistance index, was calculated by using the formula:

fasting glucose (mg dl−1)×fasting insulin(μUml−1)×405−1.

Serum chemerin concentration was determined using a commercially available ELISA kit (Biovendor, Czech). The intra-assay coefficient of variation of total cholesterol, TG, HDL-C, glucose, insulin, and chemerin were 1.1%, 1.6%, 1.9%, 1.28%, 5.3%, and 5.1% respectively.

**Statistical analysis:** Data were presented as mean ± SD. Normality assumption of the data was evaluated and was confirmed using one-sample Kolmogorov-Smirnov test in each group. Differences between normal weight and obese subjects were tested using the independent samples t-test. Changes in dependent variables resulting from the exercise intervention were assessed by two-way time-by-group repeated measures analysis of variance. The assumption of homogeneity of variances of groups was tested and confirmed by Levene test for equality of variances. Pearson's correlation was used to analyze various variables and changes in variables. *P*<0.05 was considered significant. The data were analyzed using SPSS (version 16) software.

## RESULTS

**Fig. 2:**
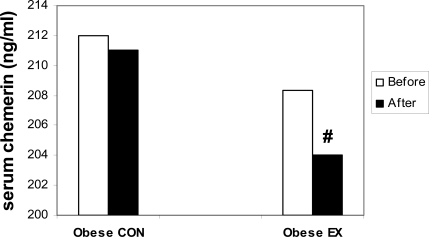
Serum chemerin levels in normal weight and obese subjects before and after 12 weeks of aerobic training (Data are expressed as the mean ± SD). ^#^ significant (*P*<0.05) difference between before and after training.

The clinical and metabolic characteristics of participants before and after 12 weeks of training are presented in table 1. Obese EX group completed at least 94% of the exercise sessions. After 12 weeks of aerobic training, the obese EX group had weight loss (*P*=0.02), which was accompanied with a significant decrease in BMI (*P*=0.01), waist circumference (WC) (*P*=0.009), percent body fat (*P*=0.03), subcutaneous fat (*P*<0.01), visceral fat (*P*=0.03), glucose (*P*=0.01), HOMA-IR (*P*=0.03), TG (*P*=0.05), TC (*P*=0.04), LDL-C (*P*=0.05), and SBP (*P*=0.04). Besides, VO_2_peak increased significantly (*P*=0.03) (Table 1) and chemerin concentrations were decreased significantly in obese EX group (*P*=0.02) (Fig. 2). Furthermore, changes in chemerin concentrations after a 12-week training period were correlated with alterations in visceral fat (*r*=0.65, *P*=0.04), subcutaneous fat (*r*=0.63, *P*=0.05) HOMA-IR (*r*=0.67, *P*=0.04), glucose (*r*=0.65, *P*=0.05), WC (*r*=0.70, *P*=0.03), and VO_2_ peak (*r*=−0.68, *P*=0.04). However, no significant changes were observed between pre- and post testing measurement of variables in the obese CON group (*P*>0.05) (Table 1).

**Table 1:** Participant characteristics before and after 12 weeks of aerobic training

Characteristics	Obese CON		Obese EX	
	before	after	before	after
**Anthropometric**				
** Weight (kg)**	96.31±6.65	96.42±6.61	97.81±5.22	94.03±4.67#
** BMI (kg/cm^2^)**	29.54±1.82	29.29±1.87	29.11±1.69	28.11±1.50#
** WC (cm)**	96.88±10.43	97.55±10.45	95.00±9.84	91.88±7.75#
** Fat (%)**	35.13±2.31	35.25±2.36	35.69±2.74	33.88±2.40#
** Visceral fat (cm^2^)**	148.60±20.68	149.00±20.69	149.45±19.75	146.63±19.06#
** Subcutaneous fat (cm^2^)**	278.40±29.45	280.10±30.31	277±28.68	272.10±24.91#
**Insulin resistance**				
** Glucose (mg dl^−1^)**	98.55±12.09	96.66±10.64	99.66±12.74	94.77±9.90#
** Insulin (µU ml**^**−1**^**)**	8.03±1.28	8.17±1.04	8.23±1.69	8.15±1.47
** HOMA-IR**	1.98±0.53	1.94±0.41	2.06±0.67	1.93±0.53#
** Lipid profile**				
** TG (mg/dl)**	120.22±26.84	122.55±27.87	118.33±26.88	116.11±27.94#
** TC (mg/dl)**	201.77±22.80	195.44±23.54	198.44±21.88	189.77±20.46#
** LDL-C (mg/dl)**	121.77±15.54	123.00±16.94	122.88±15.52	118.22±17.80#
** HDL-C (mg/dl)**	48.33±9.23	48.00±8.55	47.77±9.79	48.44±9.19
**Blood pressure**				
** SBP (mm Hg)**	130.44±7.66	129.77±7.41	131.66±6.44	129.33±7.05#
** DBP (mm Hg)**	87.77±7.69	84.22±7.62	87.22±8.59	85.11±9.22
**Vo2_max_(l/min)**	2.17±0.45	2.22±0.51	2.13±0.40	2.25±0.43#

Data are expressed as mean ± SD

# significant (*P*<0.05) difference between before and after training

BMI: body mass index/WC= waist circumference/HOMA-IR= homeostasis model assessment for insulin resistance/

TG: triglyceride/TC: total cholesterol/LDL-C: low-density lipoprotein cholesterol/HDL-C: high-density lipoprotein cholesterol/

SBP: systolic blood pressure/DBP: diastolic blood pressure/VO_2__max_: maximum oxygen consumption

There were no significant differences in the amount of calories consumed (~35 kcal.kg^−1^.d^−1^) or the percentage of calories obtained from carbohydrate (~%58), protein (~%20), and fat (~%22) between groups during the 12-wk course of aerobic training (*P*>0.05)

## DISCUSSION

The main finding of this study was that 12 weeks of aerobic training at 60–85% of HR_max_ improved insulin resistance in overweight and obese subjects. The beneficial effect of increased physical activity on insulin sensitivity was accompanied by changes in body fat (i.e., visceral fat) and circulating chemerin levels.

Adipose tissue is an active organ which secretes many proteins known as adipokines that are metabolically important^[19]^. Some of these adipokines have important functions in incidence of insulin resistance and cardiovascular complications with obesity, especially central or visceral obesity^[20]^. Chemerin is a recently discovered adipokine produced and secreted primarily by adipose tissue^[9, 10]^. In vitro studies have shown that chemerin induces insulin resistance at the levels of IRS1, Akt, and GSK3 phosphorylation and glucose uptake ^[11]^. Taken together, these results suggested that chemerin could play a role in the association between abdominal obesity and increased metabolic risk.

Physical inactivity is related to most components of the metabolic syndrome^[13]^. Regular exercise results in reduction of risk factors of obesity, type 2 diabetes, and cardiovascular disease^[14–21]^. The current study demonstrated that the obese EX group that participated in the 12-week exercise intervention improved body fatness (i.e., body weight, BMI, percent body fat, visceral fat, subcutaneous fat), insulin resistance (i.e., fasting glucose, HOMA-IR), blood lipid profile (i.e., TG, TC, LDL), and resting blood pressure (i.e., SBP) in comparison with CON group. We also found that aerobic fitness was significantly increased in the exercise group compared with the control group. Our observations are consistent with previous reports and reinforce the positive effects of exercise training on these important risk factors of metabolic syndrome, type 2 diabetes, and cardiovascular disease^[22–24]^. In the present study, 12 weeks of aerobic training decreased insulin resistance by 4.7% when comparisons were made between pre- and post intervention levels, and this is in agreement with previous investigations^[23–25]^. We observed that reduction in subjects' abdominal obesity who ran for ~60 minutes, 5d/wk is consistent with Slentz et al^[26]^ who reported that overweight and no dieting subjects performed a ~45 min activity, 4d/wk had a marked reduction in abdominal (waist circumferences) obesity. These findings confirm those previously reported from similar well controlled trials ^[27]^ and suggest that exercise without caloric restriction for ~60 minutes at 60–85% of HR_max_ is associated with reductions in abdominal obesity. Indeed, these results confirm those of previous studies that found improvement in insulin sensitivity after aerobic training and abdominal obesity reduction in obese individuals^[27]^. Several mechanisms have been proposed to be responsible for the increases in insulin sensitivity after exercise training^[16, 17]^. Changes in production of adipokines may play an important role in obesity-related cardiovascular complications and insulin resistance^[19–28]^ and it can help understand the beneficial effects of exercise.

The present study is the first to examine the influence of exercise training on chemerin serum levels. We observed a significant decrease in chemerin levels in concomitant with improvements in insulin resistance and obesity parameters after 12 weeks of aerobic training. This inverse relationship is in accordance with the theoretical negative role of chemerin in regulating insulin sensitivity and metabolic indices. It has been shown that chemerin concentrations increase with adiposity^[9–12]^. In agreement with these studies, our results showed that chemerin levels decreased significantly after body weight reduction (particularly visceral fat) in obese subjects. The decrease in chemerin concentrations indicates that changes in abdominal fat after 12 weeks of aerobic training may play an important role in the regulation of macrophage infiltration into adipose tissue and serum inflammatory markers (i.e., chemerin). Recently, surgery-induced weight loss in severely obese patients was demonstrated to reduce macrophage infiltration into adipose tissue^[29]^.

However, the present paper demonstrates that weight loss obtained through aerobic training induces a pronounced improvement in inflammation marker (chemerin) and metabolic syndrome indices. Taken together, our finding suggests that a relatively short course of training is sufficient to reduce chemerin levels, and probably, at least in part, induces early improvements in insulin resistance and other cardio metabolic risk factors after exercise related to the decrease in chemerin levels. Based on the critical important of inflammation in favoring cardiovascular and metabolic abnormalities in obesity, it could be suggested that obese subjects need to participate in aerobic training programs that lead to loss of body weight and fat reduction.

One limitation of this study was that we measured only one of the chemokines that are known to regulate inflammatory process. Clearly, other chemokines (e.g., monocyte chemoattractant protein-1) that we did not measure are also involved in the regulation of inflammation, and insulin resistance.

## CONCLUSION

A 12-week aerobic training program decreased insulin resistance and abdominal obesity, and chemerin concentrations in overweight and obese subjects. These findings suggest that exercise-induced changes in chemerin levels may be associated with the beneficial effects of exercise. Further studies are needed to elucidate the mechanisms by which exercise affects chemerin and metabolic syndrome.
